# Bag-1L Protects against Cell Apoptosis in an In Vitro Model of Lung Ischemia-Reperfusion Injury through the C-Terminal “Bag” Domain

**DOI:** 10.1155/2021/8822807

**Published:** 2021-03-17

**Authors:** Ji-ling Lv, Li-na Shi, Cong-ying Zhai, Ge-jin Wang, Yan Qu

**Affiliations:** ^1^Department of Respiratory Medicine, Shandong Second Provincial General Hospital, Jinan, 250000 Shandong, China; ^2^Department of Intensive Care Unit, Affiliated Qingdao Municipal Hospital of Qingdao University, Qingdao, 266071 Shandong, China; ^3^Zibo Traditional Chinese Medicine Hospital, Zibo, 255200 Shandong, China; ^4^Department of Respiratory Medicine, The First Hospital of Zibo, Zibo, 255200 Shandong, China; ^5^Affiliated Hospital of Zibo Vocational Institute, Zibo, 255314 Shandong, China

## Abstract

Bcl-2-associated athanogene 1 (Bag-1) is a multifunctional and antiapoptotic protein that binds to the antiapoptosis regulator Bcl-2 and promotes cell survival. To investigate the protective function of Bag-1, we examined the effects of Bag-1L, one isoform of Bag-1, in an in vitro cell culture model of lung ischemia-reperfusion injury (LIRI) generated by treatment of A549 cells with hypoxia/reoxygenation. Overexpression of full-length Bag-1L increased the viability of A549 cells and reduced cell apoptosis in response to 6 h of hypoxia/reoxygenation treatment. Furthermore, Bag-1L overexpression enhanced the heat shock protein 70 (HSP70) and Bcl-2 protein levels, increased the phosphorylation of AKT, decreased Bax and cleaved caspase-3 levels, and was able to overcome cell cycle arrest. These effects were not observed in A549 cells overexpressing a truncated form of Bag-1L lacking the “Bag domain,” denoted Bag-1L△C. The “Bag domain” is the C-terminal 47 amino acids. Taken together, the results of this study suggest that Bag-1L overexpression can protect against oxidative stress and apoptosis in an in vitro LIRI model, with a dependence on the Bag domain.

## 1. Introduction

Lung ischemia-reperfusion injury (LIRI) can occur in many clinical conditions such as resuscitation from circulatory arrest, lung transplantation, and trauma [1, 2]. LIRI can cause pulmonary edema and acute respiratory distress syndrome [3] through different mechanisms [4, 5], and apoptosis has been shown to play a critical role in the complex pathophysiological process in LIRI [6].

Bcl-2-associated athanogene 1 (Bag-1) is a multifunctional and antiapoptotic protein that binds Bcl-2 to promote cell survival [7, 8]. Bag-1 is encoded by the Bag-1 gene, and via different translation initiation sites, four isoforms of Bag-1 are produced, including Bag-1L (52 kDa), Bag-1 (34 kDa), Bag-1 M (46–48 kDa), and Bag-1S (29 kDa) [7]. In human cells, Bag-1L is constitutively located in the nucleus, while Bag-1M is partitioned between the cytoplasm and the nucleus and is not expressed in all species. Bag-1S is preferentially localized in the cytoplasm [9, 10], but cellular stress causes relocation of cytoplasmic Bag-1S to the nucleus. Interestingly, both BAG-1S and Bag-1L suppress heat shock-induced apoptosis to the same extent, suggesting a critical role for these isoforms in the nucleus [11]. All Bag-1 isoforms share a highly conserved protein interaction region near the C terminus, which is called the “Bag domain.” The Bag domain binds and regulates the heat shock protein 70 (HSP70)/heat shock cognate 70 (HSC70) molecular chaperones, and this activity is closely related to its antiapoptotic function [12]. HSP70 has been shown to effectively protect alveolar epithelial cells against apoptosis and ameliorate the injury caused by LIRI [13]. Site-directed mutagenesis of the Bag domain reduces binding to HSP70 and Bag-1 activity in vitro [12, 14, 15]. Therefore, it is speculated that Bag-1/HSP70 interaction may also regulate the antiapoptosis effect of Bag-1 in LIRI through the Bag domain.

Our previous research showed that silencing of HSP70 increases apoptosis in LIRI through the inhibition of the extracellular signal-regulated kinase (ERK) and protein kinase B (AKT) signaling pathways [16]. The phosphoinositide 3-kinase (PI3K)/AKT signaling pathway is involved in several cellular processes such as cell apoptosis, proliferation, and transcription [17–20]. The RAF/MEK/ERK pathways, also known as the mitogen-activated protein kinase (MAPK) module, are the classical pathways of the MAPK family, and this kinase cascade is activated after ischemia/reperfusion in many organs [21–23].

To better understand the protective function of Bag-1L, in the present study, we investigated the effect of Bag-1L in an in vitro cell-based model of LIRI as well as the role of the Bag domain in the observed antiapoptotic activity. For this purpose, we generated A549 cells overexpressing full-length Bag-1L or Bag-1L△C (truncated Bag-1L lacking the C-terminal 47 amino acids). Cells overexpressing full-length Bag-1L were also characterized, and their susceptibility to apoptosis was compared with that of cells expressing Bag-1L△C. We also examined the effects of Bag-1L on the expression of apoptosis-related protein (Bax, Bcl-2, and caspase-3) in the LIRI model. Furthermore, we explored whether Bag-1 modulates the response to LIRI through activation of the MAPK/ERK and PI3K/AKT pathways.

## 2. Materials and Methods

### 2.1. Cell Line and Culture

The A549 cell line, derived from a human alveolar cell carcinoma, is the most widely used in vitro models for type II pulmonary alveolar epithelial cells (ATII cells) [24]. Cardella et al. successfully established an I/R cell culture model of clinical lung transplantation using A549 cells [25]. The A549 cell line was provided by the Teaching and Research Section of Microbiology of Qingdao University Medical College (Qingdao, People's Republic of China), and the use of this cell line was approved by the institutional review board of Qingdao University. A549 cells were cultured in Dulbecco's modified Eagle's medium (DMEM, Hyclone, USA) supplemented with 10% fetal bovine serum (Hyclone), penicillin (100 U/ml), and streptomycin (100 *μ*g/ml). The cells were maintained in a humidified atmosphere with 5% CO_2_ at 37°C, and the culture media was replaced every 2–3 days.

### 2.2. Bag-1 Adenovirus and Cell Infection

Five groups of cells were prepared: the recombinant full-length Bag-1L adenovirus infection group (Bag-1L group), Bag-1L adenovirus control group (Bag-1L control group), recombinant Bag-1L△C adenovirus infection group (Bag-1L△C group), Bag-1L△C adenovirus control group (Bag-1L△C control group), and noninfection group.

A549 cells were infected by adenovirus containing Bag-1L cDNA or Bag-1L△C DNA (Shanghai Genechem Company, China) according to the manufacturer's instructions. Briefly, A549 cells were seeded in 96-well plates at a density of 5 × 10^3^ cells per well and incubated at 37°C in 5% CO_2_ overnight. When the cells reached 50–70% confluency, the DMEM was discarded, and the cells were washed once with phosphate-buffered saline (PBS). For the Bag-1L group, 100 *μ*l cryopreserved recombinant adenovirus suspension, including 20 *μ*l adenovirus liquid, 70 *μ*l enhanced infection solution, and 10 *μ*l polybrene (5 *μ*g/ml), was added directly to each well. For the Bag-1L△C group, a volume of 40 *μ*l mixture including 14 *μ*l adenovirus, 26 *μ*l DMEM, and 10 *μ*l polybrene was added for incubation for 4 h, and then another 40 *μ*l mixture including 30 *μ*l DMEM and 10 *μ*l polybrene was added for incubation at 37°C for 12 h. After the cells were incubated at 37°C for 48 h, DMEM with 1 *μ*g/ml puromycin selective medium was added, according to optimization based on the toxicity curve. Every 2–3 days, the selection medium was changed for a total of 10–14 days. The cells were then maintained in the selective medium containing 0.5 *μ*g/ml puromycin for 1 month.

### 2.3. Hypoxia/Reoxygenation Model

To create a LIRI cell model, we subjected the A549 cells to simultaneous shortages of nutrients and oxygen, according to a method described previously [26, 27]. Briefly, the culture medium was removed under sterile conditions, and serum-free, low-glucose DMEM was added immediately for all groups. The cells in 25 cm^2^ cell culture flasks (Corning, USA) were then placed in an airtight jar with one Anaero-Indicator and one pouch of AnaeroPack for culture at 37°C. The concentration of oxygen was adjusted to 1% or less, and when the Anaero-Indicator changed to pink, we started timing hypoxia from 1 h [28]. Hypoxia was stopped by opening the airtight jar after 6 h, and the cells were recultured in normal DMEM and culture conditions for 24 h to complete the reoxygenation model.

### 2.4. qRT-PCR

Total RNA was extracted by TRIzol reagent (Invitrogen, USA) according to the manufacturer's instructions and quantified by UV spectrophotometry (Implen, Germany). cDNA was synthesized to serve as the PCR template using the M-MLV Reverse Transcriptase Kit (Roche, Switzerland) according to the manufacturer's protocol. The cDNA was mixed with FastStart Essential DNA Green Master Mix (Roche). The primers for Bag-1L were 5′-CTTCATGTTACCTCCCAGCA-3′ and 5′-ACGGTGTTTCCATTTCCTTC-3′, and the glyceraldehyde-3-phosphate dehydrogenase (GAPDH) primer sequences were 5′-ATCTCTGCCCCCTCTGCTGA-3′ and 5′-GATGACCTTGCCCACAGCCT-3′. The amplification was performed using a Light Cycler 96 (Roche) according to the manufacturer's protocol. Each sample was run three times. The relative levels of Bag-1L were calculated using the 2^-*ΔΔ*Ct^ method.

### 2.5. Western Blotting

Western blotting was performed according to previously described methods (Hu et al. 2013). Briefly, A549 cells were collected and washed three times with ice-cold PBS after hypoxia/reoxygenation treatment for 6 h. Cellular protein was extracted by lysate containing 200 *μ*l ice-cold radioimmunoprecipitation assay (RIPA) lysis buffer, 2 *μ*l ice-cold phenylmethylsulfonyl fluoride (PMSF) lysis buffer, and 2 *μ*l ice-cold phosphatase inhibitors for 30 min. The supernatant fraction was collected after the mixture was centrifuged for 15 min at 12000 rpm. The concentration of proteins was determined using a BCA kit (TransGen Biotech, China), and the proteins were denaturated at 100°C for 5 min. The sample proteins were subjected to 10% sodium dodecyl sulfate- (SDS-) polyacrylamide gel electrophoresis (PAGE) and blotted onto a polyvinylidene difluoride (PVDF) membrane. The primary antibodies used in the experiment were anti-Bag-1 antibody (1 : 1000, Cell Signaling Technology, USA), anti-HSP70 antibody (1 : 1000, Abcam, USA), anti-AKT antibody (1 : 10,000, Abcam), anti-p-AKT antibody (1 : 5000, Abcam), ant-ERK antibody (1 : 1000, Abcam), anti-p-ERK antibody (1 : 1000, Abcam), and anti-*β*-actin antibody (1 : 1000, Abcam). The membranes were then incubated with the secondary antibodies (goat anti-mouse for Bag-1 and goat anti-rabbit for the others) for 2 h at room temperature. The results were observed with the Fusion FX Imaging System (Vilber Lourmat, France), and the gray-scale values were analyzed using the Bio-Rad Quantity One software (Bio-Rad, USA).

### 2.6. Cell Viability Assay

Cell viability was measured using the Cell Counting Kit-8 (Boster, China). Briefly, 5 × 10^3^ cells/well were seeded into 96-well plates and incubated for 24 h. The cells were treated with hypoxia for 2, 4, 6, 8, 10, 12, or 24 h, followed by reoxygenation for 24 h. Subsequently, 10 *μ*l CCK-8 mixed with 90 *μ*l DMEM was added to each well and incubated for 1 h. Finally, the absorbance of the solution in each well was measured at 450 nm by a microplate reader (Bio-Tek, USA). Samples of 100 *μ*l DMEM were used as the blank control. The mean values from triplicate experiments were analyzed.

### 2.7. Flow Cytometric Apoptosis Assay

A549 cells were seeded in 6-well plates at a density of 2 × 10^5^ cells/well and incubated for 24 h. After 6 h of hypoxia/reoxygenation treatment, the cells were harvested using trypsin without EDTA and resuspended in 1× binding buffer to a concentration of 1–5 × 10^6^ cells/ml. Annexin V-APC and propidium iodide (PI) from an apoptosis detection kit (eBioscience, USA) were used for cell staining according to the manufacturer's protocol. Apoptotic cells were quantified with a FACStar Plus flow cytometer (Becton Dickinson, USA). The data were analyzed using the BD Accuri C6 software. Mean values from triplicate experiments were analyzed.

### 2.8. Lactate Dehydrogenase (LDH) Activity Assay

To measure LDH activity, five parallel wells were set for each experimental group. After hypoxia/reoxygenation for 6 h, the culture media from each group were collected for detection of LDH activity using an LDH activity assay kit (Nanjing Jian Cheng Biotechnology, China) according to the manufacturer's protocol. Each sample was run in triplicate.

### 2.9. Cell Cycle Distribution Analysis by Flow Cytometry

A549 cells were seeded in 6-well plates at a density of 2 × 10^5^ cells/well and incubated for 24 h. After hypoxia/reoxygenation treatment for 6 h, according to the manufacturer's recommendations for a PI flow cytometry kit (BD, USA), the cells were washed and incubated with trypsin for 3 min at 37°C, before fixation with 66% ethanol on ice. Next, 1 × 10^6^ cells per group were stained with PI (5% PI in PBS, containing 0.5% RNase A) and subjected to DNA content analysis using the FACStar Plus flow cytometer. The mean values of three independent experiments were analyzed using FlowJo.

### 2.10. Statistical Analysis

All values were expressed as mean ± standard deviation (SD) values, and differences among groups were analyzed with the SPSS 17.0 statistical software. One-way analysis of variance (ANOVA) followed by Tukey's test was used to identify the differences in multiple comparisons. *p* < 0.05 was considered indicative of a statistically significant difference.

## 3. Results

### 3.1. Overexpression of Bag-1L and Bag-1L△C in A549 Cells

**Figure 1:**
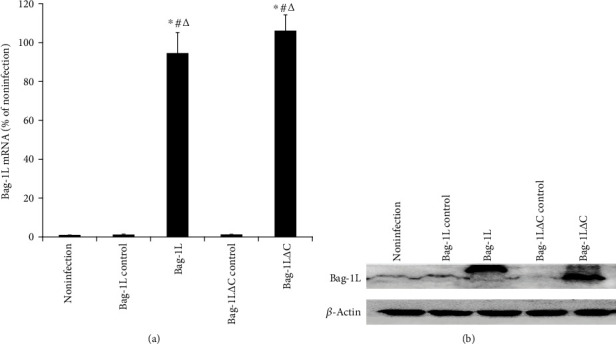
Detection of Bag-1L in each group after adenovirus infection for 48 h. A dramatic increase in Bag-1L (a) mRNA and (b) protein levels in both the Bag-1L and Bag-1L△C groups compared with the respective adenovirus control groups and the noninfection group. ∗*p* < 0.01 vs. the noninfection group; ^#^*p* < 0.01 vs. the Bag-1L control group; ^∆^*p* < 0.01 vs. the Bag-1L△C control group.

After 48 h of infection, the real-time PCR results showed a dramatic increase in Bag-1L mRNA expression in both the Bag-1L and Bag-1L△C groups compared with the adenovirus control groups and the noninfection group (Figure 1(a), all *p* < 0.01). Western blot analyses also confirmed that exogenous Bag-1L (e-Bag-1L) and Bag-1L△C (e-Bag-1L△C) proteins were expressed in the respective adenovirus infection groups (Figure 1(b)).

### 3.2. Effects of Bag-1L and Bag-1L△C Expression on A549 Cell Viability

CCK-8 assay was used to evaluate the viability of A549 cells. After exposure to hypoxia-reoxygenation for different times, the highest viability of A549 cells was observed after 6 h of treatment, and therefore, hypoxia for 6 h was chosen for our further research. The increased proliferation of A549 cells after 6 h of hypoxia may be explained by previous observations that a short period of hypoxia stimulates cell proliferation, whereas long-term hypoxia reduces cell activity [29, 30].

**Figure 2:**
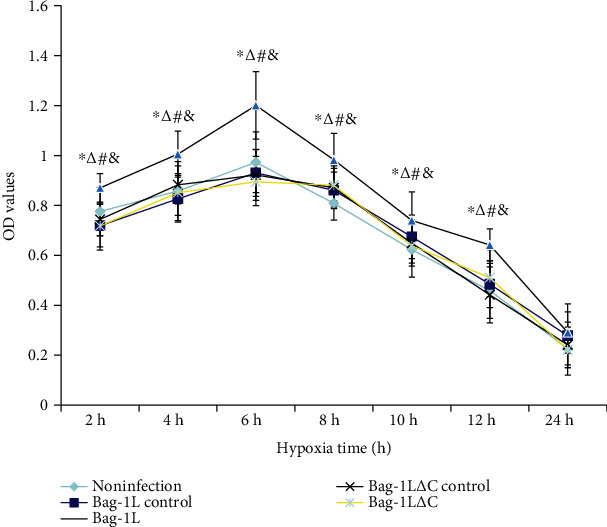
Viability of A549 cells in different groups after hypoxia/reoxygenation for different durations. ∗*p* < 0.01 vs. the noninfection group; ^∆^*p* < 0.05 vs. the Bag-1L△C control group; ^#^*p* < 0.05 vs. the Bag-1L control group; ^&^*p* < 0.05 vs. the Bag-1L△C group.

To assess whether overexpression of Bag-1L has an effect on A549 cell viability after exposure to hypoxia-reoxygenation and whether the effect of Bag-1L is dependent on its C-terminal, A549 cells were infected with Bag-1L or Bag-1L△C for 48 h and then treated with hypoxia-reoxygenation for an additional 6 h. Overexpression of Bag-1L△C in A549 cells did not increase cell survival rates, whereas overexpression of full-length Bag-1L resulted in significantly increased cell viability compared with that of the Bag-1L control group and noninfection group (both *p* < 0.01, Figure 2). No difference in cell viability was observed between the adenovirus control group and noninfection group (*p* > 0.05, Figure 2).

### 3.3. Effects of Bag-1L and Bag-1L△C on A549 Cell Apoptosis and LDH Release

**Figure 3:**
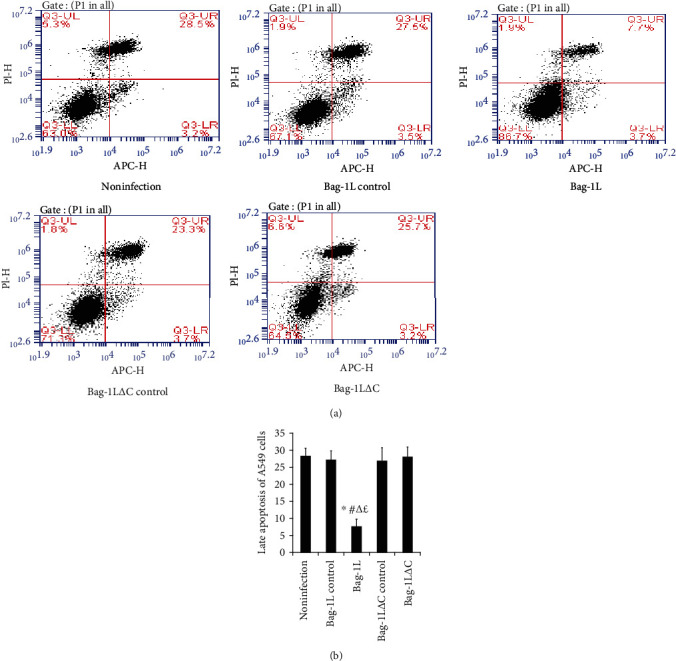
Apoptosis of A549 cells after hypoxia/reoxygenation. After treatment with hypoxia/reoxygenation for 6 h, overexpression of Bag-1L significantly increased the late apoptosis of A549 cells. (a) Representative results of Annexin V-APC/PI staining of A549 cells exposed to hypoxia/reoxygenation for 6 h obtained via flow cytometry. *Q3-UL* necrosis, *Q3-UR* late apoptosis, *Q3-LR* early apoptosis, and *Q3-LL* viable cells. (b) Data analyses of late apoptotic cells in each group. ∗*p* < 0.01 vs. the noninfection group; ^#^*p* < 0.01 vs. the Bag-1L control group; ^∆^*p* < 0.01 vs. the Bag-1L△C control group; ^£^*p* < 0.01 vs. the Bag-1L△C group.

The results of flow cytometric analyses revealed increased apoptosis of A549 cells at 6 h after hypoxia/reoxygenation in each group. Overexpression of Bag-1L but not Bag-1L△C significantly attenuated the apoptotic rate of the cells compared with the rates in the adenovirus control group and noninfection group (Figures 3(a) and 3(b), *p* < 0.05). Moreover, the apoptotic rates in the adenovirus control group and noninfection group were not significantly different (Figures 3(a) and 3(b), *p* > 0.05).

**Figure 4:**
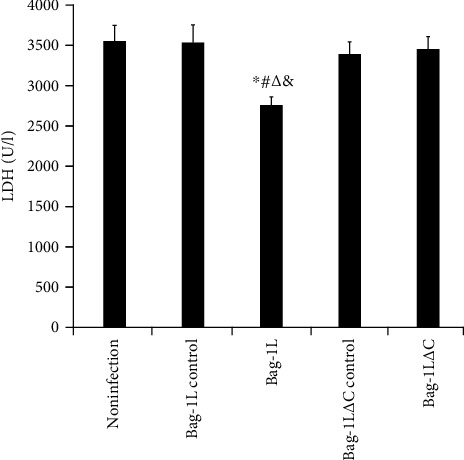
Measurement of LDH activity in the culture medium. ∗*p* < 0.01 vs. the noninfection group; ^#^*p* < 0.01 vs. the Bag-1L control group; ^∆^*p* < 0.01 vs. the Bag-1L△C control group; ^&^*p* < 0.01 vs. the Bag-1L△C group.

Bag-1L-overexpressing cells attenuated the release of LDH after context of hypoxia/reoxygenation for 6 h compared with the control cells (Figure 4, *p* < 0.01). Overexpression of Bag-1L△C however did not result in altered LDH release in the same conditions compared to the adenovirus control group and noninfection group (Figure 4, *p* > 0.05).

### 3.4. Effects of Bag-1L and Bag-1L△C on Cell Cycle Distribution

**Figure 5:**
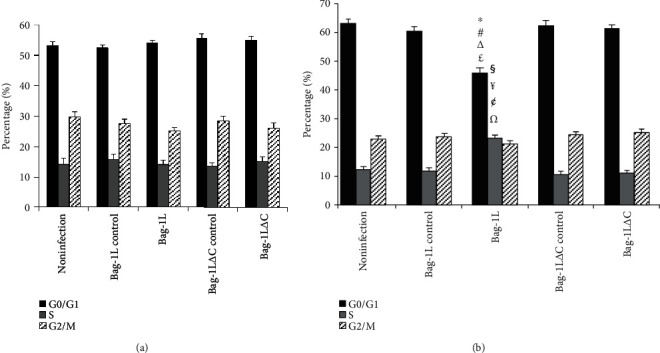
Overexpression of Bag-1L altered the cell cycle distribution of A549 cells in response to hypoxia/reoxygenation. (a) After infection for 48 h, there were no differences in each phase of the cell cycle between groups with overexpression of Bag-1L or Bag-1L△C. (b) Hypoxia/reoxygenation decreased significantly the percentage of cells in G0/G1 phase and increased the percentage of cells in S phase in the Bag-1L group. ∗*p* < 0.01 vs. the G0/G1 phase in the noninfection group; ^#^*p* < 0.01 vs. the G0/G1 phase in the Bag-1L control group; ^∆^*p* < 0.01 vs. the G0/G1 phase in the Bag-1L△C control group; ^£^*p* < 0.01 vs. the G0/G1 phase in the Bag-1L△C group; ^§^*p* < 0.01 vs. the S phase in the noninfection group; ^¥^*p* < 0.01 vs. the S phase in the Bag-1L control group; ^¢^*p* < 0.01 vs. the S phase in the Bag-1L△C control group; *^Ω^p* < 0.01 vs. the S phase in the Bag-1L△C group.

After adenovirus infection for 48 h, no change in cell cycle distribution was observed among the groups of A549 cells (Figure 5(a), *p* > 0.05). However, after treatment with hypoxia/reoxygenation for 6 h, the percentage of cells at G0/G1 phase in the Bag-1L group was significantly lower, and the percentage of cells at S phase was significantly increased compared with the respective percentages in the Bag-1L△C group, adenovirus control group, and noninfection group (Figure 5(b), *p* < 0.01).

### 3.5. Effects of Bag-1L and Bag-1L△C on Regulation of Gene Expression

**Figure 6:**
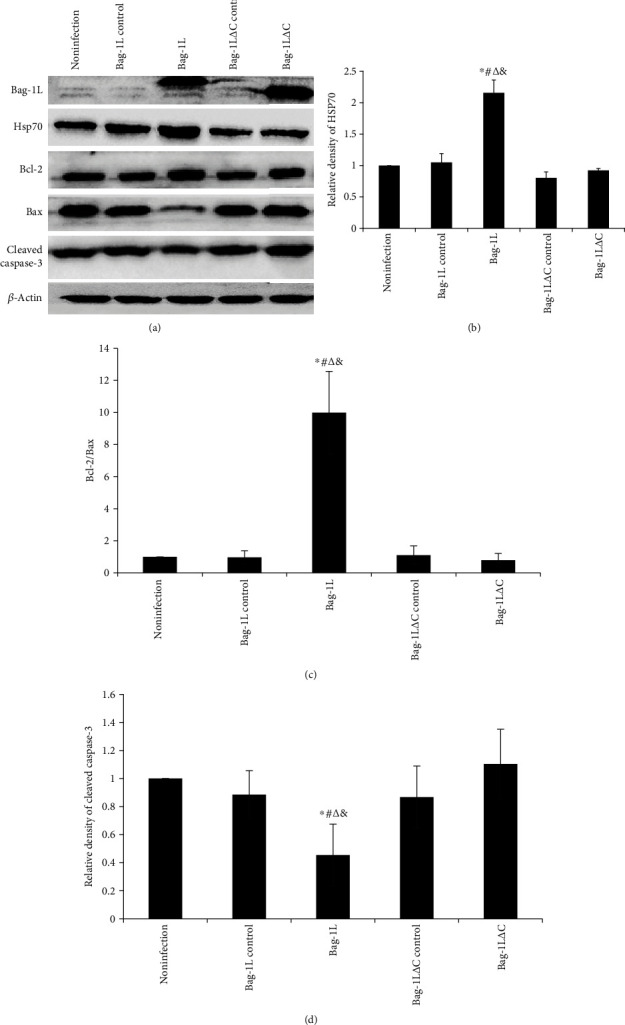
Expression levels of Bag-1L, HSP70, Bcl-2, Bax, and cleaved caspase-3 upon hypoxia/reoxygenation. (a) Western blots results for HSP70, Bcl-2, Bax, and cleaved caspase-3. Relative protein expression according to the optical density ratios of (b) HSP70 and (c) Bcl-2/Bax relative to *β*-actin: ∗*p* < 0.01 vs. the noninfection group; ^#^*p* < 0.01 vs. the Bag-1L control group; ^∆^*p* < 0.01 vs. the Bag-1L△C control group; ^&^*p* < 0.01 vs. the Bag-1L△C group. (d) Optical density ratio for cleaved caspase-3 relative to *β*-actin: ∗*p* < 0.05 vs. the noninfection group; ^#^*p* < 0.05 vs. the Bag-1L control group; ^∆^*p* < 0.05 vs. the Bag-1L△C control group; ^&^*p* < 0.05 vs. the Bag-1L△C group.

After treatment with hypoxia/reoxygenation for 6 h, Bag-1L and Bag-1L△C protein levels were upregulated in the adenovirus infection group compared with these levels in the adenovirus control group and noninfection group (Figure 6(a)). Bag-1L overexpression resulted in increased endogenous HSP70 (Figures 6(a) and 6(b), *p* < 0.01) and Bcl-2 protein levels and decreased Bax (Figures 6(a) and 6(c), *p* < 0.01) and cleaved caspase-3 levels (Figures 6(a) and 6(d), *p* < 0.05) compared with the levels of these proteins in the other groups.

**Figure 7:**
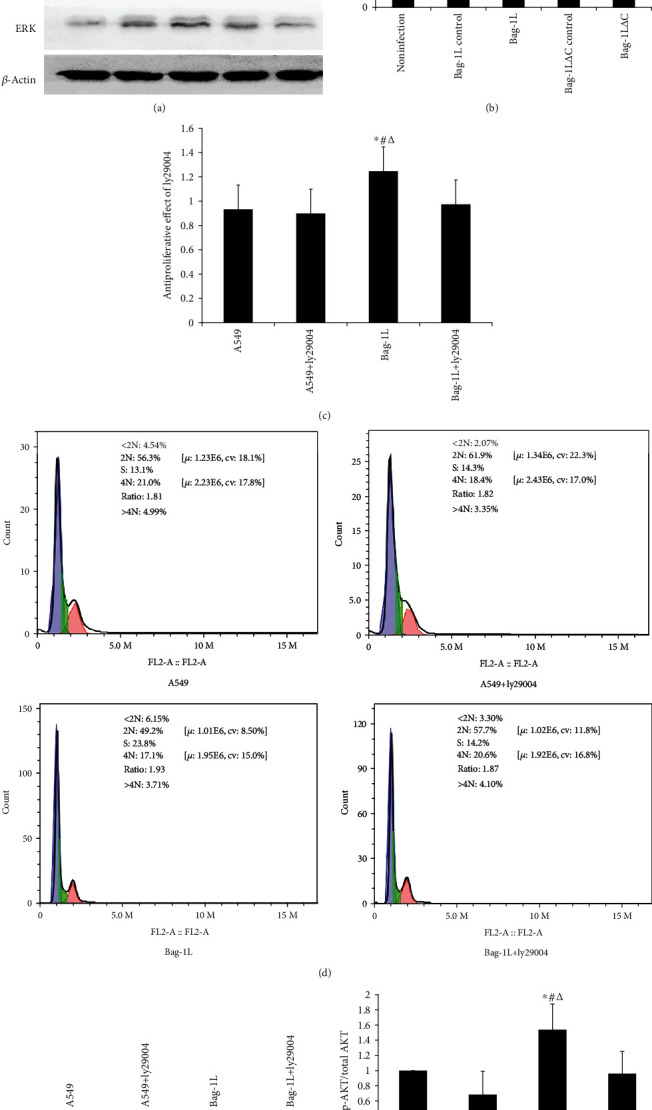
Effects of Bag-1L on AKT, p-AKT, ERK, and p-ERK protein expression levels after exposure to hypoxia/reoxygenation for 6 h. (a) Western blot results for AKT, p-AKT, ERK, and p-ERK. (b) Relative protein expression based on the optical density ratio relative to that for *β*-actin. Data are presented as the ratio of p-AKT/total AKT. ∗*p* < 0.01 vs. the noninfection group; ^#^*p* < 0.01 vs. the Bag-1L control group; ^∆^*p* < 0.01 vs. the Bag-1L△C control group; ^&^*p* < 0.01 vs. the Bag-1L△C group. (c) Cell proliferation with addition of ly29004 (5 *μ*mol/l): *p* < 0.01. (d) Ly294002 altered the cell cycle distribution. (e, f) Phosphorylation of AKT was inhibited by ly29004. Three independent experiments were performed. ∗*p* < 0.01 vs. the A549 group; ^#^*p* < 0.01 vs. the A549+ly29004 group; ^∆^*p* < 0.01 vs. the Bag-1L+ly29004 group.

After all group of cells were exposed to hypoxia/reoxygenation for 6 h, the AKT, p-AKT, ERK, and p-ERK protein levels were detected by western blot assay. The results showed that the total AKT and Erk protein levels were not changed, but the p-AKT protein level was increased in the Bag-1L group (Figures 7(a) and 7(b), *p* < 0.01). Unexpectedly, the phosphorylation levels of Erk were elevated both in the Bag-1L group and Bag-1L△C group (Figure 7(a)). To confirm whether the PI3K/AKT pathway mediates the effects of Bag-1L, we treated cells overexpressing Bag-1L with ly29004 (which targets PI3K/AKT) for 4 h before hypoxia/reoxygenation treatment. Then we investigated the effects of Bag-1L and the specific pharmacological inhibitor, ly29004, on the proliferation of A549 cells. Treatment with ly29004 (5 *μ*mol/l) inhibited the proliferation of cells overexpressing Bag-1L (Figure 7(c), *p* < 0.01) and altered the percentage of cells in cell cycle arrest (Figure 7(d)). Additionally, we investigated the inhibitory effects of ly29004 on signaling protein expression. The phosphorylation of AKT produced by Bag-1L was inhibited by ly29004 significantly (Figures 7(e) and 7(f)).

## 4. Discussion

LIRI is a disease caused by ischemia and reperfusion in the vascular tissues and organs. In the pathological process, the reperfusion results in serious damage to tissues and cells. It has been well established that LIRI induces cell death via the process of apoptosis [31, 32], indicating that prevention of apoptosis may be a potential strategy for LIRI treatment.

An in vitro model of LIRI was achieved by simultaneous shortage of nutrients and oxygen in cultured cells, and the dysregulation of cell viability showed the successful construction of the in vitro LIRI injury model. The results of the present study demonstrated that Bag-1L can protect alveolar cells from apoptosis induced by simulated ischemia/reperfusion, and this is consistent with previous research showing that Bag-1 can suppress apoptosis in diverse systems [33]. The present study also provides the first analyses of Bag-1L function in LIRI and the mechanism of action through an evaluation of the effects of a specific mutation.

The Bcl-2 family includes antiapoptotic genes such as Bak, Bcl-2, and Bcl-xL [34] and proapoptotic genes such as Bad and Bax [35]. Bcl-2 family members have been shown to prevent apoptosis in lung epithelial cells [36, 37], and it is well known that Bcl-2 can bind to Bax to form Bcl-1/Bax heterodimers to regulate apoptosis [38–40]. The ratio of Bcl/Bax determines the survival of cells in ischemic disease; in other words, when Bcl is dominant, cells survive [41, 42]. At the same time, caspase-3 is a member of the caspase family and one of the key executors of apoptosis, with previous research showing that activation of caspase-3 is important for apoptosis following ischemia [43]. Bcl-2 acts upstream to prevent caspase activation [44]. In our study, overexpression of Bag-1L decreased cellular apoptosis while also increasing Bcl-2/Bax expression and decreasing cleaved caspase-3 expression. Our findings support that Bag-1L, but not Bag-1L△C, alleviates the apoptosis of LIRI by modulating the Bax/Bcl-2 ratio and preventing caspase-3 activation.

LDH is a glycolytic enzyme present in many cells. Hypoxia/reoxygenation can enhance glycolysis and increase intracellular LDH. Ischemia/reperfusion can damage the physiological function of cells and then produce a series of pathological and biochemical changes, such as increased membrane permeability and extracellular release of LDH from damaged cells, which is a sensitive reflection of cell activity and function. Therefore, the LDH concentration can be measured to indirectly evaluate the degree of cell damage and necrosis. Our previous study showed that hypoxia/reoxygenation leads to an increase in the intracellular LDH level [16]. A decreased LDH level in the Bag-1L group, but not in the Bag-1L△C group, further supports the role of Bag-1L in preserving the stability of the cell membrane. These results suggest that Bag-1L, but not Bag-1L△C, alleviates cell injury and apoptosis in the model of hypoxia-reoxygenation.

Previous studies showed Bag-1 provides protection against neuronal apoptosis through upregulation of HSP70 [45, 46], and our study also revealed increased expression of HSP70 in the Bag-1L group. Because Bag-1L binds tightly to HSP70 chaperones, we assume Bag-1L stabilizes HSP70 protein, leading to HSP70 accumulation in cells. Overexpression of HSP70 effectively protects cells against apoptosis and antagonizes the effects of LIRI [13, 47], whereas silencing HSP70 makes cells more vulnerable to apoptosis in LIRI [16]. Prior research showed that Bag-1 functions as a cochaperone of HSP70/HSC70 proteins and modulates their activity in many important cellular processes by binding to the ATPase domain [48]. In contrast, Bag-1L△C was shown to be unable to bind to HSP70 or to affect the folding or refolding activity [48, 49]. Another previous study demonstrated that binding to chaperones is essential for Bag-1-mediated cytoprotection from apoptosis induced by ischemia-reperfusion [11]. In the current study, A549 cells that overexpressed Bag-1L△C remained sensitive to apoptotic stimuli induced by hypoxia/reoxygenation and did not increase the HSP70 level. Thus, it is likely that Bag-1L protection of A549 cells against hypoxia/reoxygenation is correlated with its ability to activate the HSP70 chaperone activity.

In addition to the interaction of HSP70 with Bag-1L, the Bag domain also mediates interaction with RAF-1 [50]. The binding of HSC70/HSP70 and RAF-1 is competitive [15], and therefore, we checked whether Bag-L△C, like Bag-1L, still induces Raf-1-mediated Erk phosphorylation. Stimulation by hypoxia/reoxygenation markedly increased Erk phosphorylation in the Bag-1L group. However, unexpectedly, overexpression of Bag-1L△C also affected Erk phosphorylation. Our results are consistent with previous reports [49, 51] suggesting that Raf-1 kinase may be a promising focus for future experiments to further elucidate the pathways modulated by Bag-1L.

The PI3K/AKT pathway is active in a wide variety of cells, and previous studies showed activation of the PI3K/AKT signaling pathway alleviates the apoptosis induced by ischemia-reperfusion injury [52–55]. In the present study, we observed increased Act pathway activation based on phosphorylation of the AKT pathway proteins in the Bag-1L group but not in the Bag-1L△C group. In addition, treatment with ly294002, a synthetic PI3K inhibitor, significantly reduced the levels of phosphorylated AKT as well as cell proliferation in the Bag-1L group. These results suggest that Bag-1L may inhibit the cell apoptosis induced by LIRI via PI3K/Akt activation.

Notably, apoptosis is related to cell cycle arrest [56]. Given that the PI3K/AKT signaling pathway plays an important role in proliferation, Myklebust et al. showed that ly294002 can inhibit proliferation via induction of cell cycle arrest in the G0/G1 or G2/M phases of the cell cycle [57, 58], and we also examined the effects of Bag-1L expression on cell cycle progression. The current study revealed that overexpression of Bag-1L resulted in S arrest in A549 cells following hypoxia/reoxygenation, while ly294002 changed the cell cycle distribution. Thus, we propose that the mechanism by which overexpression of Bag-1L protects A549 cells from apoptosis may involve cell cycle arrest, further confirming that the PI3K/AKT pathway is involved in the process.

## 5. Conclusions

In summary, overexpression of Bag-1L resulted in a potent antiapoptotic action in an in vitro LIRI model, and this action was partly dependent on the “Bag” domain of Bag-1, through HSP70 binding and activation of the p-AKT/AKT signaling pathway. It is conceivable that small-molecule drugs that mimic Bag-1L and occupy the binding site for HSP70 could have cytoprotective properties and offer promise as potential therapeutics for LIRI treatment. However, more studies in vivo are needed to confirm the effects of Bag-1L in LIRI in the future.

## Data Availability

The datasets generated and analyzed during the present study are available from the corresponding author on reasonable request.
